# Synthesis and Characterization of Polynorbornenes with Nitronaphthyl Side‐Chains

**DOI:** 10.1002/marc.202500381

**Published:** 2025-08-25

**Authors:** Gero Bramlage, Alina Kasberg, Guillaume Delaittre

**Affiliations:** ^1^ Institute of Organic Chemistry and Macromolecular Chemistry Heinrich Heine University Düsseldorf Düsseldorf Germany; ^2^ Organic Functional Molecules Organic Chemistry University of Wuppertal Wuppertal Germany; ^3^ Macromolecular Chemistry I University of Bayreuth Bayreuth Germany

**Keywords:** fluorescence, nitration, quenching, ring‐opening metathesis polymerization

## Abstract

Three new norbornene monomers carrying naphthyl‐based sidechains with varying levels of electron deficiency are synthesized, characterized by ^1^H NMR, 2D‐NMR, UV–vis, and fluorescence spectroscopies, as well as mass spectrometry. The kinetics of their ring‐opening polymerization using a third‐generation Grubbs catalyst are established, and the resulting polymers are characterized by size‐exclusion chromatography, along with UV–vis and fluorescence spectroscopies. These naphthalene‐based polynorbornenes are shown to exhibit fluorescence, accompanied by a quenching behavior when exposed to an electron‐rich aromatic compound.

## Graphical abstract

Fluorescent polynorbornenes incorporating naphthyl ester side‐chains with various degrees of nitration are synthesized by ring‐opening metathesis polymerization. Their potential for the quantitative detection of electron‐rich aromatic compounds is demonstrated.

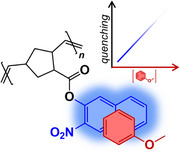

## 1 Introduction

Chemical sensing by fluorescence is an attractive way of providing chemical information about the composition of a sample. Its major advantages over classical analytical methods lie in its rapid responsiveness [1, 2] and high sensitivity, even allowing for the detection of single molecules [3, 4, 5], making it a valuable tool for monitoring in environmental, clinical, or industrial settings. Due to the variety of analytes, matrices, and required sensitivity levels, many forms of fluorescence quenching‐based sensors have been developed, such as carbon nanodots for the detection of heavy metals in water [6, 7, 8], metal organic frameworks responding to nucleic acids [9, 10, 11, 12] or modified gold nanoparticles for the detection of lead [13], thiols [14], or hemoglobin [15]. Polymers have also been used in sensing systems to stabilize nanoparticles [16] but also as probes [17, 18, 19], particularly in the case of conjugated polymers [20]. An interesting aspect of the use of polymers is the possibility to form porous networks, which are insoluble and have a very high surface area, making them easier to handle compared to suspended nanodots or metallic nanoparticles [21].

Intermolecular interactions between a fluorescent probe and its analyte are essential for efficient detection via fluorescence quenching [22, 23]. Among these interactions, π–π stacking plays a particularly significant role, especially when there is a pronounced discrepancy in electron density between the interacting species [24]. The nitro group, being one of the most potent electron‐withdrawing substituents [25], substantially lowers the electron density of aromatic rings, thereby enhancing their ability to engage in π–π stacking with electron‐rich fluorophores. This characteristic not only facilitates effective quenching but also explains the widespread use of nitroaromatic compounds as quenchers in fluorescence‐based sensing systems [26, 27, 28]. While nitroaromatics are commonly studied as analytes due to their toxic, explosive, and environmentally persistent nature [29], their strong electron‐deficient π systems also render them valuable for the detection of electron‐rich aromatic compounds.

In this work, we designed a nitrated, fluorescent polyaromatic system that is stabilized as part of a polymer and can be used for the detection of electron‐rich compounds by quenching. We synthesized and characterized the monomers, investigated the polymerization reactions, and examined the optical properties of the polymers as well as their responsiveness toward quenching agents.

## 2 Results and Discussion

**SCHEME 1:**
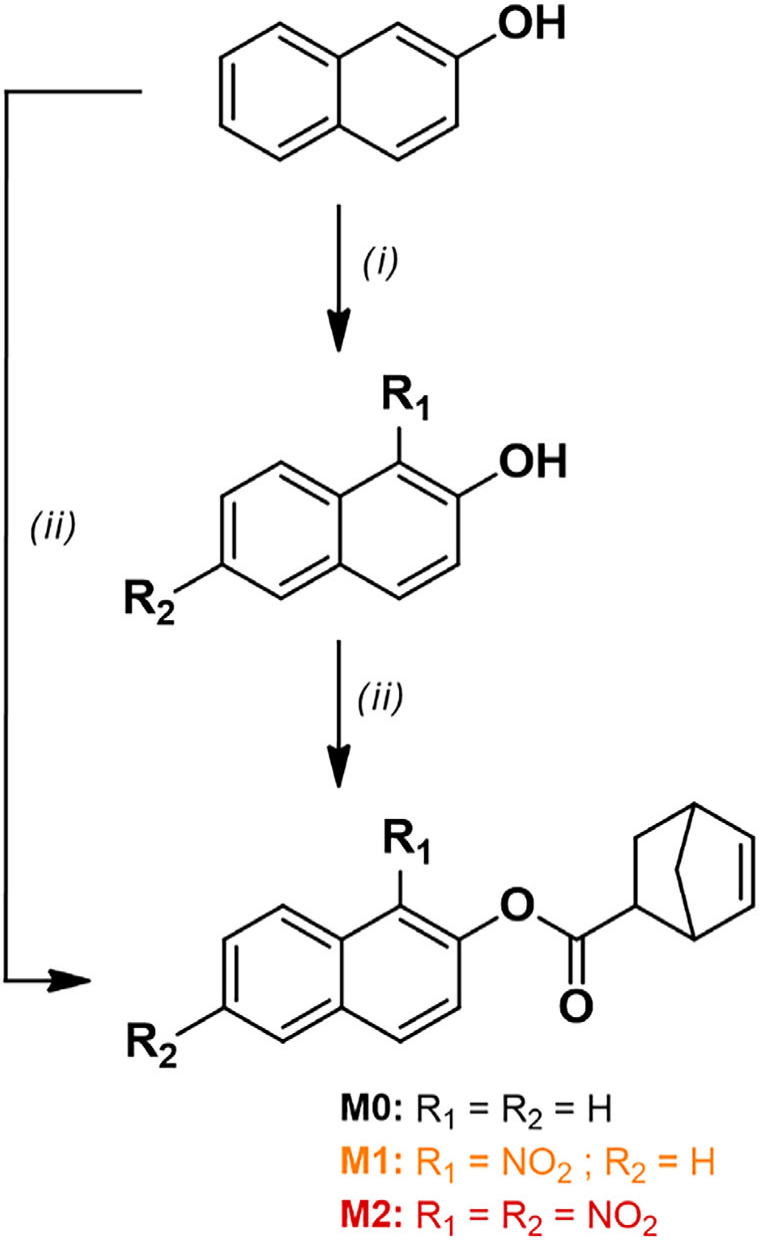
Reaction scheme for synthesis of monomers **M0**, **M1**, and **M2**. (i) V_2_O_5_, H_2_SO_4_/HNO_3_; (ii) 5‐norbornene‐2‐carboxylic acid, EDC/DMAP, DCM, 0°C → RT.

The initial design included the (nitrated) naphthalene motif appended with a methacrylate ester as a polymerizable unit via esterification of naphthalen‐2‐ol derivatives with methacrylic anhydride. However, free‐radical polymerization of the nitrated monomers was difficult, with monomer conversions not exceeding 10 mol% in the best case, even in the presence of cyclodextrins as possible complexing agents (data available upon request). This confirms prior observations that nitroaromatic compounds retard and even inhibit radical polymerization [30, 31, 32], even though it has been postulated that methacrylates should be less affected [33]. Therefore, similarly to previous studies on the design of polymers with nitroaromatic or radical trapping groups in their side‐chains [34, 35], we resorted to ring‐opening metathesis polymerization (ROMP). To this aim, norbornene derivatives with pendant naphthyl esters were synthesized (Scheme 1). The nitrated precursors to monomers **M1** and **M2** were synthesized from naphthalen‐2‐ol according to a reported nitration protocol [36] and purified by column chromatography. The success and the extent of the nitration were assessed by ^1^H NMR spectroscopy and mass spectrometry, while the positions of the nitro groups were determined using 2D NMR spectroscopy. The polymerizable norbornene group was connected to naphthalen‐2‐ol and its nitrated derivatives by activating 5‐norbornene‐2‐carboxylic acid using 1‐ethyl‐3‐(3‐dimethylaminopropyl)carbodiimide (EDC) in the presence of 4‐dimethylaminopyridine (DMAP), with overall yields ranging from 35% (**M2**) to 48% (**M1**) and 93% (**M0**). The monomers were solid at ambient temperature and soluble in classic solvents for ROMP such as dichloromethane (DCM), *N*,*N*‐dimethylacetamide (DMAc), and *N*,*N*‐dimethylformamide (DMF). The UV–vis spectra of the monomers are very similar to those of their corresponding naphthalen‐2‐ol derivatives, enabling some general statements (Figure S3): i) Nitration produces an ipsochromic shift of the main UV absorption peak – minor for the mononitrated compounds but more significant for the dinitrated ones; ii) while mononitration leads to a broad and weak absorption from this main peak until almost 450 nm, dinitration leads to a series of better defined peaks in the same region, and iii) esterification of the naphthalen‐2‐ols produces an ipsochromic shift of the entire spectrum leading to a compaction of the absorption below 400 nm.

Synthesis of the corresponding polynorbornenes was carried out by ROMP at room temperature in *N*,*N*‐dimethylacetamide (DMAc) with a 3‐bromopyridine‐based Grubbs III ruthenium catalyst (G3) at a monomer concentration of 0.5 M. Pyridine (10 eq. relative to G3) was added to tame the reactivity of G3 and retard secondary metathesis [37]. Monomer conversion was monitored by ^1^H NMR spectroscopy and macromolecular growth by size‐exclusion chromatography (SEC) in DMAc. The conversion could be followed by the disappearance of the norbornene olefinic proton signals in the ^1^H NMR spectrum around 6.5–6.3 ppm, and concomitant appearance of a broad signal at 5.9–5.4 ppm corresponding to the main‐chain unsaturations.

**FIGURE 1:**
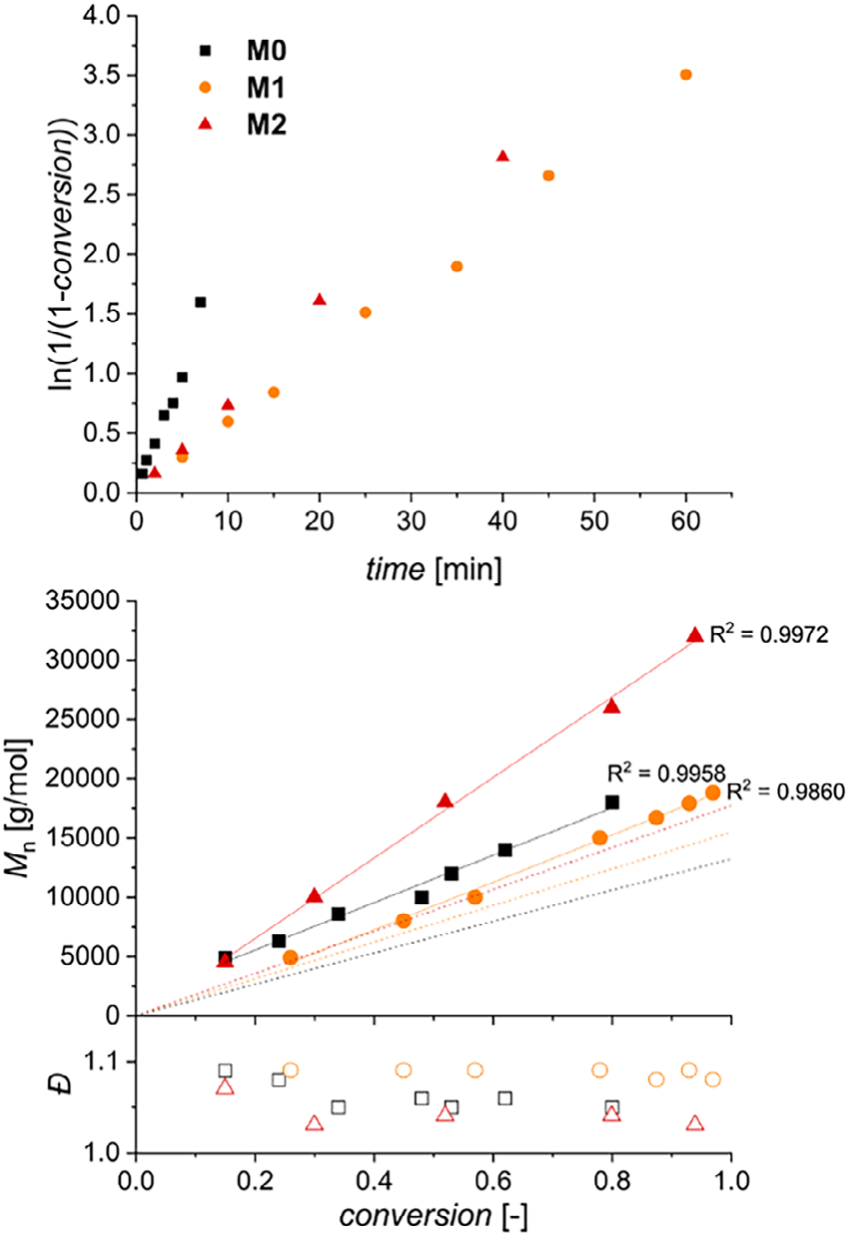
Kinetic and macromolecular data for the ROMP of monomers **M0**, **M1**, and **M2** (DMAc, RT, [M] = 0.5 mol L^−1^, [M]:[G3]:[pyridine] = 50:1:10): (top) semilogarithmic kinetic plot; (bottom) *M*_n_ (plain symbols) and *Ð* (hollow symbols) evolution with conversion. Solid lines (linear fit) and dashed lines (evolution of theoretical *M_n_*).

Figure 1 compiles the semi‐logarithmic plots obtained by aliquoting the reaction mixtures at regular time intervals. All three monomers exhibit first‐order kinetics with respect to the monomer concentration, indicating a constant concentration in active species and providing a first hint at a controlled polymerization. Despite the addition of pyridine as well as the low reaction temperature and monomer concentration, **M0** polymerizes relatively fast, reaching 80% conversion in less than 10 min. **M1** and **M2** propagate significantly slower, yet do reach 80% conversion in short timespans, that is, 30 and 20 min, respectively. While the steric effects around the reactive norbornene double bonds should not be substantially altered by nitration, additional chelation of the Ru center with nitro groups may occur. However, a doubling in the concentration of nitro groups – from **M1** to **M2** – does not result in a decrease of the polymerization rate but rather in a slight increase. Importantly, number‐average molar masses increase linearly with conversion and dispersity values remaining between 1.0 and 1.1 in all cases, hinting further at a controlled process occurring exclusively by propagation.

**FIGURE 2:**
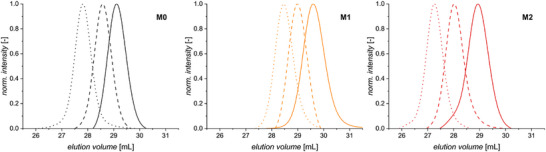
Size‐exclusion chromatograms in DMAc of polymers obtained by ROMP of **M0**, **M1**, and **M2** with [M]:[G3] ratios of 50 (—), 100 (‐ ‐ ‐), and 150 (···). (DMAc, RT, [M] = 0.5 mol L^−1^, [G3]:[pyridine] = 50:1:10).

Further proof of the controlled polymerization was established by varying the [M]:[G3] with all other conditions remaining the same. Figure 2 demonstrates the possibility to target various molar masses while keeping a narrow molar mass distribution. The trends in calibration‐based molar masses observed in Figure 1, that is, the largest values for **M2** and the lowest values for **M1** are here confirmed.

A batch synthesis with [M]:[G3] = 50 and otherwise the same experimental conditions was performed to produce **P0**, **P1**, and **P2** from **M0**, **M1**, and **M2**, respectively, and investigate their thermal properties. Decomposition and glass‐transition temperatures are collated in Table 1.

**TABLE 1:** Decomposition temperature (*T*_d_) and glass‐transition temperature (*T*_g_) and for polynorbornenes **P0**, **P1**, and **P2**.

	*T*_d_ [°C]	*T*_g_ [°C]
P0	250	91
P1	230	89
P2	112	78

A higher degree of nitration clearly leads to a decrease in thermal stability, with a halving of the decomposition temperature for dinitration. This result is expected, as nitrated aromatics are known to be thermally unstable [38] and the release of N_2_ gas is a strong thermodynamic driving force [39]. The trend of a decreasing *T_g_* with increasing nitration degree is unexpected, since the higher polarity should lead to a stronger intermolecular interaction; however, there are also examples pointing to the contrary, as the melting point of anthracene (216°C) [40] is higher than that of 9‐nitroanthracene (141°C) [41]. We nevertheless postulate that the thermal stability of **P2** would be sufficient in sensing contexts.

**FIGURE 3:**
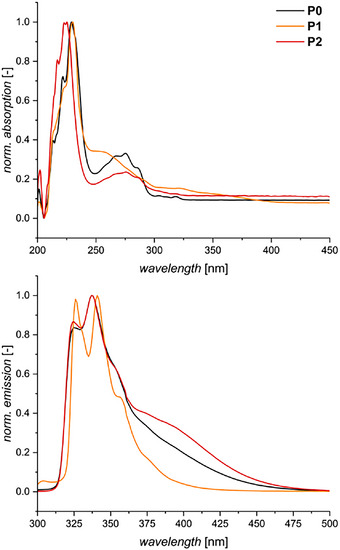
Normalized absorption (top) and fluorescence (bottom) spectra of **P0**, **P1**, and **P2**.

Furthermore, as the naphthyl low‐molar‐mass compounds exhibited some distinct absorption patterns in the UV–vis range, the polymers were also investigated for their photophysical properties, including fluorescence. The absorption spectra of the polymers exhibit less obvious differences than their monomer counterparts (Figure 3, top). The ipsochromic shift of the main absorption peak is only noticeable for the dinitrated polymer **P2**, yet is relatively small. A broad and weak absorption until approx. 400 nm is still visible for the mononitrated motif (**P1**). Yet, in the spectral range from 250 to 400 nm, no significant difference is observed between the non‐nitrated and the dinitrated polymers, **P0** and **P2**, respectively.

To obtain the fluorescence spectra, excitation at 270 nm was performed (Figure 3, bottom). In analogy to their absorption properties, the fluorescence behaviors of **P0** and **P2** are relatively similar, with only slightly more emitted radiation in the 370–500 nm range. **P1**, however, stands out with a distinctly narrower emission range. In all cases, a multicomponent signal is emitted, indicating the occurrence of several relaxation pathways, often observed in polycyclic aromatics due to vibrational sublevels [42].

Finally, all three polymers were tested for their quenching activity toward electron‐rich aromatics. This compound class was selected since a strong attractive force between electron‐rich quenchers and the electron‐deficient side‐chains can be presumed. As model compounds, anisole, toluene, and pyridine were chosen. All polymers were dissolved in HPLC‐grade tetrahydrofuran (THF) at a concentration of 0.1 mg mL^−1^ and exposed to an excitation wavelength of 270 nm, with the quenching agent in THF being added stepwise.

**FIGURE 4:**
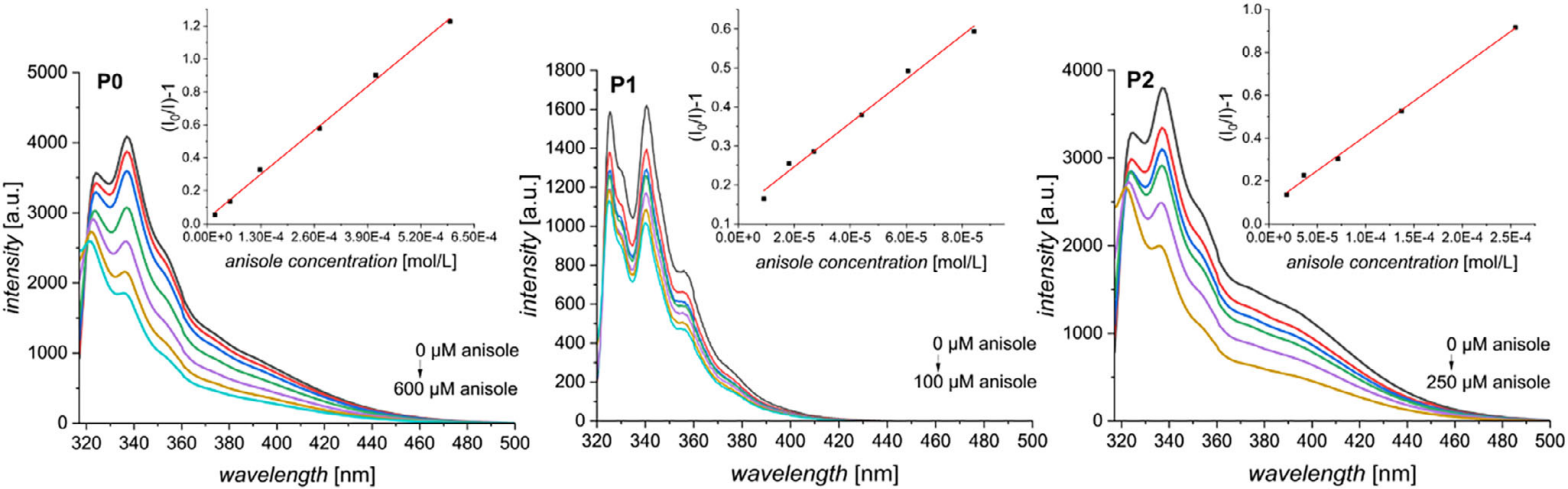
Fluorescence measurements for **P0**, **P1**, and **P2** with increasing quencher concentrations and corresponding Stern‐Volmer‐Plots with linear fit (insert).

From the fluorescence emission stacks (Figure 4 and Figures S4–S6), it is visible that quenching does not affect all radiative transitions in a concerted manner. Indeed, the emission intensity at 337 nm is dropping off more quickly than the one at 325 nm.

Comparing the intensity *I* of the peak at 340 nm at various quencher concentrations to the original intensity without quencher (*I*_0_) leads to the establishment of so‐called Stern‐Volmer‐plots ((*I*_0_/*I*) – 1 = f(*[quencher]*)) (see insets in Figure 4). Gratifyingly, at this wavelength, we observe a linear dependence on the quencher concentration, which is a great advantage for sensing applications. The slope of each plot corresponds to the Stern‐Volmer constant *K*_SV,_ which is equal to the product of the quenching rate constant (*k*_q_) and the lifetime of the emissive excited state of the naphthyl derivative without quenching agent present (*τ*_0_). For **P0**, **P1**, and **P2**, *K*_SV_ are compiled in Table 2.

**TABLE 2:** Stern–Volmer constants for polymer/quencher systems in M^−1^.

	Anisole	Toluene	Pyridine
P0	2050	172	3285
P1	5625	22	6104
P2	3247	45	4748

To obtain the absolute quenching rate constants, time‐resolved fluorescence spectroscopic measurements would be necessary. Nevertheless, *K*_SV_ values indicate that – surprisingly – mononitrated **P1** would be a more sensitive fluorophore than the dinitrated species in two of the three tested cases. However, it is also known that a too high *K*_SV_ leads to a short dynamic range and possibly to unwanted sensitivity to background quenchers. Therefore, **P1** and **P2** would certainly find their way in specific cases.

## 3 Conclusion

Three new norbornene monomers with naphthyl ester side‐chains incorporating 0, 1, or 2 nitro groups were synthesized and converted to functional polynorbornenes by ring‐opening polymerization mediated by a third‐generation Grubbs catalyst. The addition of pyridine to the polymerization helped tame the fast kinetics and achieve low dispersity. Increasing degrees of nitration led to polymers with significantly lower thermal stability and slightly lower glass‐transition temperatures. The naphthyl‐based polynorbornenes were shown to be fluorescent and were subsequently tested for their response toward an electron‐rich quencher. They all showed fluorescence quenching behavior with a linear response to the quencher concentration, a pre‐requisite for sensing applications. Future directions may lead to extended polyaromatic systems (anthracene and pyrene) with various degrees of nitration, as well as combinations with electron‐rich counterparts in order to produce donor–acceptor systems. To investigate the fluorescence (quenching) behavior, notably to examine the possible formation of excimers, which typically possess longer fluorescence lifetimes, and to determine quenching rate constants, time‐resolved fluorescence spectroscopy should be performed. The occurrence of excimers should also be more simply probed through copolymerization with non‐functional norbornenes, inducing variable spacing and modulating interaction between fluorophores. Additionally, more elaborate topologies could be explored, that is, cross‐linked networks or particles, to increase the stability and practicability of the system.

## Conflicts of Interest

The authors declare no conflicts of interest.

## Supporting information




**Supporting file 1**: marc70024‐sup‐0001‐SuppMat.docx.

## Data Availability

The data that support the findings of this study are available from the corresponding author upon reasonable request.
