# Supplementary material for: Synthesis and Characterization of Polynorbornenes with Nitronaphthyl Side‐Chains
Source: Macromol Rapid Commun. 2025 Aug 25;46(23):e00381. doi: 10.1002/marc.202500381 (PMC12687714; doi:10.1002/marc.202500381)
Supplement: Supplementary file 1 — Supporting file 1: marc70024‐sup‐0001‐SuppMat.docx. [file MARC-46-e00381-s001.pdf]

Supporting Information

Synthesis and Characterization of Polynorbornenes with Nitronaphthyl Side-Chains

*Gero Bramlage, Alina Kasberg, Guillaume Delaittre**

1 Experimental Part

1.1 Chemicals and Glassware

9-Oxo-9H-fluorene-4-carboxylic acid (98 %, abcr), vanadium(V) oxide (VWR), nitric acid (68 %, Acros), sulfuric acid (95-97 %, VWR), 1,1'-carbonyldiimidazole (CDI, Alfa Aesar), naphthalen-2-ol (Sigma), triethylamine (Sigma), oxiran-2-ylmethyl methacrylate (Aldrich), bicyclo[2.2.1]hept-5-ene-2-carboxylic acid (Sigma), DMAP (≥99 %, Roth), EDC · HCl (Roth), [1,3-bis-(2,4,6-trimethylphenyl)-2-imidazolidine-yliden]-dichloro-(phenylmethylen)-bis-(3-brompyridin)-ruthenium(II) (Grubbs III, abcr), pyridine (≥99 %, Fisher Chemicals), anisole (≥99 %, Roth), DCM (≥99 %, Fisher), DMF (≥99 %, Fisher), DMAc (≥99 %, Fisher), DCM-d2 (deutero), and DMF-d7 (deutero) were used as received Unless otherwise stated, all reactions were performed under inert gas and in flame-dried glassware, using Schlenk techniques.

1.2 Characterization

All NMR measurements were performed on either a BRUKER AVANCE 400 spectrometer at 400.13 MHz or a BRUKER AVANCE III 600 spectrometer at 600.13 MHz. All NMR measurements were obtained at room temperature. Chemical shifts (δ) were measured in ppm (parts per million) relative to the reference signal of tetramethyl silane (TMS: $\delta = 0.00$ ppm). Deuterated chloroform ($CDCl_3$), dimethylformamide (DMF-d7) and dimethyl sulfoxide (DMSO-d6) were used as solvents for the measurements. Their residual solvent resonance signals (^{1}H-NMR: δ ($CHCl_3$) = 7.26 ppm, δ (DMF) = 8.02 ppm, δ (DMSO) = 2.50 ppm) served as references for the chemical shift, multiplicity (s – singlet, d – doublet, t – triplet, q – quartet, m – multiplet), coupling constant (e.g., ^3J [Hz]), and integration.

All mass spectrometry measurements were performed on a Bruker micrOTOF coupled to an Agilent 1100 Series liquid chromatography (LC) instrument. Electrospray ionization (ESI) method was used for ionization.

Thin-layer chromatography (TLC) was conducted with precoated glass-backed plates (silica gel) and visualized by exposure to UV light (254 nm) or by staining with a basic potassium permanganate ($KMnO_4$) solution and subsequent heating.

For the chromatographic column purifications, silica gel from Carl Roth (particle size 0.035–0.070 mm) was used.

All SEC measurements were performed on a Polymer Standards Service PSS/Agilent SECurity^2-GPC using DMAc + LiBr (0.28 g/L) at 50 °C as eluent. The system comprised an autoinjector, a PSS PFG precolumn (7 µm, 8 × 50 mm), three separating PFG columns (7 µm, 8 × 300 mm, porosities: 100, 1000, 4000 Å), and a refractive-index detector (RID). The SEC system was calibrated with linear poly(methyl methacrylate) standards and the samples were injected with a maximum concentration of 2 g L^{-1}.

TGA was performed on a Mettler Toledo TGA/DSC 1 STAR^e System with a SDTA sensor in the range of 35–900 °C, a heating rate of 10 K min^{-1}, and an argon stream of 50.0 mL min^{-1}.

DSC measurements were carried out on a Mettler Toledo TGA/DSC 1 STAR^e System (FRS5 400W) with a heating rate of 10.0 K min^{-1} and nitrogen stream of 30.0 mL min^{-1}. The investigated temperature range was between –50 °C and 130 °C. To determine the T_g, the infliction point of the second or third out of three heating curves was used.

UV-Vis spectroscopic measurements were performed using a Mettler Toledo UV5.

Fluorescence measurements were performed using a Jasco FP-8350 spectrofluorometer or a Hitachi fluorescence spectrophotometer F-7000 at an excitation wavelength of 270 nm, with a Xe light source excitation and emission bandwidth of 10 nm and a scan speed of 100 nm min^{-1}.

1.3 Synthesis

1.3.1 Naphthalen-2-yl bicyclo[2.2.1]heptane-2-carboxylate (**M0**)

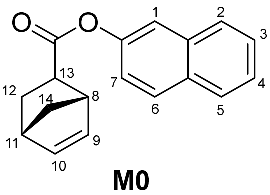

M0

Bicyclo[2.2.1]hept-5-ene-2-carboxylic acid (2.60 mL, 21.3 mmol, 1.0 eq), naphthalen-2-ol (3.00 g, 20.8 mmol, 1.0 eq), and DMAP (0.5911 g, 4.165 mmol, 0.2 eq.) were dissolved in DCM (84 mL) and a solution of EDC· HCl (4.0219 g, 31.274 mmol, 1.5 eq.) in DCM (36 mL) was added slowly at 0 °C. The solution was stirred overnight at room temperature. The reaction mixture was washed three times with water (30 mL) and brine (30 mL). The organic layer was dried over $MgSO_4$, the solid was filtered off, the solvent was removed *in vacuo* and the reaction crude was purified using column chromatography yielding a white solid (5.0960 g, 18.9 mmol, 91%)..

$\boldsymbol{R_f}$ **(DCM)** = 0.33.

^{1}H NMR (600 MHz, $CDCl_3$): *δ* [ppm] = 7.82 (tt, J = 17.8 Hz, J = 8.5 Hz, 3H, H 2, H 6, H 5), 7.52 – 7.42 (m, 2H, H 3, H 4), 7.21 (ddd, J = 35.8 Hz, J = 8.8 Hz, J = 2.3 Hz, 2H, H 7, H 1), 6.30 (dd, J = 5.8 Hz, J = 3.1 Hz, 1H, H 9), 6.15 (dd, J = 5.8 Hz, J = 2.8 Hz, 1H, H 10), 3.44 (d, J = 4.4 Hz, 1H, H 11), 3.28 (dt, J = 8.9 Hz, J = 3.7 Hz, 1H, H 13), 3.01 (d, J = 8.9 Hz, 1H, H 8), 1.68 – 1.37 (m, 4H, H 15, H 14).

^{13}C NMR (151 MHz, $CDCl_3$): *δ* [ppm] = 173.43, 148.62, 138.36, 138.26, 135.71, 133.81, 132.24, 131.38, 129.31, 127.76, 127.61, 126.48, 125.58, 121.23, 118.42, 49.81, 46.90, 46.46, 46.02, 43.75, 43.45, 42.74, 41.81, 30.63, 29.46.

LC-MS (ESI): *m*/*z* ($C_{18}H_{17}O_2$): $[M+H]^+$ found: 265.1303, calculated: 265.1273.
m/*z* ($C_{18}H_{20}NO_2$): $[M+NH_4]^+$ found: 282.1574, calculated: 282.1538.

1.3.2 1-Nitronaphthalen-2-ol (**1**)

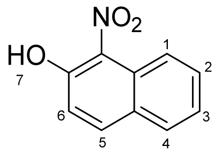

1

In a 1 L flask, naphthalen-2-ol (3.00 g, 20.80 mmol, 1.0 eq.) and vanadium(V) oxide (V_2O_5, 0.57 g, 3.12 mmol, 0.15 eq.) were suspended in DCM (180 mL). After adding nitric acid (68 %, 0.87 mL, 21 mmol, 1.0 eq.) the mixture was placed in an ultrasonic bath at room temperature for 30 min. The reaction mixture was washed four times with water (150 mL). The combined aqueous layers were reextracted three times with DCM (200 mL). The organic layers were combined and dried over $MgSO_4$. The solvent was removed *in vacuo* to give a dark red viscous oil (3.54 g). The crude product was purified using column chromatography (gradient: DCM/ethyl acetate) to give an orange solid (1.92 g, 10.2 mmol, 49 %).

***R*$_f$ (DCM)** = 0.40.

^{1}H NMR (600 MHz, $CDCl_3$): *δ* [ppm] = 12.18 (s, 1H, H 7), 8.93 (d, J = 8.8 Hz, 1H, H 1), 8.01 (d, J = 9.0 Hz, 1H, H 5), 7.82 (d, J = 8.0 Hz, 1H, H 4), 7.74 (ddd, J = 8.7 Hz, J = 7.0 Hz, J = 1.5 Hz, 1H, H 2), 7.51 (dd, J = 8.1 Hz, J = 6.9 Hz, 1H, H 3), 7.29 – 7.22 (m, 1H, H 6).

^{13}C NMR (151 MHz, $CDCl_3$): *δ* [ppm] = 158.99, 139.35, 131.13, 129.47, 128.86, 127.11, 125.80, 123.42, 119.55.

MS (ESI): *m*/*z* ($C_{10}H_6NO_3$): $[M–H]^-$ found: 188.0353, calculated: 188.1627.

1.3.3 1,6-Dinitronaphthalen-2-ol (**2**)

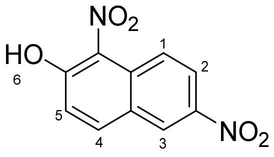

2

In a 1 L flask, naphthalen-2-ol (3.00 g, 20.80 mmol, 1.0 eq.) and vanadium(V) oxide (V_2O_5, 0.57 g, 3.12 mmol, 0.15 eq.) were suspended in DCM (180 mL). After adding nitric acid (68 %, 0.87 mL, 21 mmol, 1.0 eq.) the mixture was placed in an ultrasonic bath at room temperature for 90 min. The reaction mixture was washed four times with water (150 mL). The combined aqueous layers were reextracted three times with DCM (200 mL). The organic layers were combined and dried over $MgSO_4$. The solvent was removed *in vacuo* to give a dark red viscous oil (3.54 g). The crude product was purified using column chromatography (gradient: DCM/ethyl acetate) to give an orange solid (2.53 g, 10.8 mmol, 52 %).

***R*f (DCM)** = 0.10.

^{1}H NMR (600 MHz, $CDCl_3$): δ [ppm] = 12.19 (s, 1H, H 6), 9.07 (d, J = 9.6 Hz, 1H, H 1), 8.75 (d, J = 2.4 Hz, 1H, H 3), 8.48 (dd, J = 9.6 Hz, J = 2.4 Hz, 1H, H 2), 8.18 (d, J = 9.1 Hz, 1H, H 4), 7.46 (d, J = 9.1 Hz, 1H, H 5).

^{13}C NMR (151 MHz, $CDCl_3$): δ [ppm] = 160.66, 144.90, 139.76, 130.46, 127.90, 125.10, 124.19, 122.32, 53.56.

MS (ESI): *m*/*z* ($C_{10}H_5N_2O_5$): $[M–H]^-$ found: 233.0369, calculated: 233.1627.

1.3.4 1-Nitronaphthalen-2-yl-bicyclo[2.2.1]hept-5-ene-2-carboxylate (**M1**)

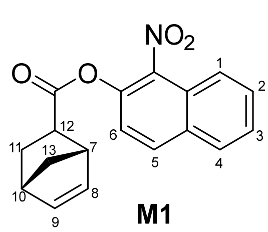

M1

Bicyclo[2.2.1]hept-5-ene-2-carboxylic acid (0.65 mL, 5.3 mmol, 1.0 eq), **1** (1.00 g, 5.27 mmol, 1.0 eq.) and DMAP (0.1499 g, 1.056 mmol, 0.2 eq) were dissolved in dry DCM (28 mL). At 0°C a solution of EDC · HCl (1.0256 g, 7.9751 mmol, 1.5 eq.) in dry DCM (12 mL) was added over 10 min. The reaction was stirred overnight at room temperature. The reaction was washed three times with water (30 mL) and brine (30 mL). The organic layer was dried over $MgSO_4$, the solid was filtered off, and the solvent was removed *in vacuo* to give an orange oil (1.62 g). The crude product of was purified by column chromatography (gradient: cyclohexane/DCM) to give an orange solid (1.60 g, 5.17 mmol, 98 %).

***R*f (DCM)** = 0.50.

^{1}H NMR (600 MHz, $CDCl_3$): δ [ppm] = 8.08 – 7.87 (m, 3H, H 5, H 4, H 1), 7.74 – 7.60 (m, 2H, H 3, H 2), 7.38 – 7.27 (m, 1H, H 5), 6.39 – 6.13 (m, 2H, H 9, H 8), 3.44 (s, 1H, H 10), 3.30 (dt, J = 8.7 Hz, J = 3.8 Hz, 1H, H 12), 3.03 (s, 1H, H 7), 1.62 – 1.51 (m, 4H, H 15, H 13).
LC-MS (ESI): *m/z* ($C_{18}H_{19}N_2O_4$): $[M+NH_4]^+$ found: 327.1351, calculated: 327.3538.

1.3.5 1,6-Dinitronaphthalen-2-yl (1S,4S)-bicyclo[2.2.1]hept-5-ene-2-carboxylate (**M2**)

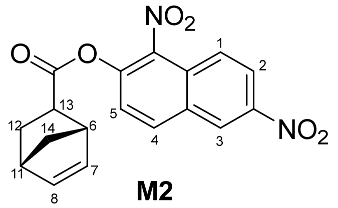

M2

Bicyclo[2.2.1]hept-5-ene-2-carboxylic acid (0.52 mL, 4.3 mmol, 1.0 eq), **1** (1.00 g, 4.28 mmol, 1.0 eq.) and DMAP (0.12 g, 0.86 mmol, 0.2 eq) were dissolved in dry DCM (28 mL). At 0°C a solution of EDC · HCl (0.8256 g, 6.40 mmol, 1.5 eq.) in dry DCM (12 mL) was added over 10 min. The reaction was stirred overnight at room temperature. The reaction was washed three times with water (30 mL) and brine (30 mL). The organic layer was dried over $MgSO_4$, the solid was filtered off, and the solvent was removed *in vacuo* to give an orange solid (1.42 g). The crude product of was purified by column chromatography (gradient: cyclohexane/DCM) to give an orange solid (1.01 g, 2.86 mmol, 67 %).
$\boldsymbol{R_f}$ **(DCM)** = 0.30.
^{1}H NMR (400 MHz, $CDCl_3$): δ [ppm] = 8.88 (dd, J = 5.2 Hz, J = 2.3 Hz, 1H, H 3), 8.43 (ddd, J = 9.4 Hz, J = 4.1 Hz, J = 2.3 Hz, 1H, H 4), 8.30 – 8.18 (m, 1H, H 1), 8.03 (ddt, J = 14.0 Hz, J = 9.4 Hz, J = 0.8 Hz, 1H, H 2), 7.55 (dd, J = 18.9 Hz, J = 9.0 Hz, 1H, H 5), 6.36 – 6.05 (m, 2H, H 8, H 7), 3.46 – 3.39 (m, 1H, H 12), 3.30 (dt, J = 9.2, 3.9 Hz, 1H, H 13), 3.02 (s, 1H, H 6), 2.07 (dtd, J = 12.9 Hz, J = 8.8 Hz, J = 3.9 Hz, 1H, H 12), 1.62 – 1.34 (m, 5H, H 15, H 14).
LC-MS (ESI): *m/z* ($C_{18}H_{14}N_2O_6Na$): $[M+Na]^+$ found: 377.0766, expected: 377.3092.

1.4 Polymerizations

All polymerization reactions were performed at room temperature (air conditioned at 23 °C) in dry, degassed DMAc, under argon atmosphere, using a monomer concentration of 0.5 M. The concentration of the 3rd generation Grubbs M300 catalyst was varied to achieve the desired degree of polymerization and 10 eq. of pyridine in reference to the concentration of the catalyst were added to control the reaction rate. To the solution of the monomer and pyridine in DMAc, a solution of catalyst in DMAc was added to initiate the reaction. An excess of ethyl vinyl ether was added to quench the reaction. To obtain polymers for thermal, UV-Vis and fluorescence measurements, the reaction time was adjusted depending on the monomer to achieve a conversion of around 90%, in accordance to previous kinetic

studies. The reaction solution was added to cold methanol to precipitate the polymer. If necessary, the polymer was dissolved in DCM and again precipitated from cold methanol.

1.5 Fluorescence quenching

Polymers **P0**, **P1**, and **P2** with a DP of 50 were dissolved in HPLC grade THF at a concentration of 0.1 mg mL^{-1} and a fluorescence spectrum was recorded with an excitation wavelength of 270 nm. A solution of quenching agent in THF was added stepwise and a fluorescence spectrum was recorded after each addition. The fluorescence intensity was adjusted to the increasing volume by assuming a linear relationship between concentration and fluorescence intensity. Stern-Volmer-plots were created in Origin 2022 V 9.9.0.225 by plotting the maxima of the fluorescence spectrum against the concentration of the quencher and fitting it linearly.

2 Additional Data

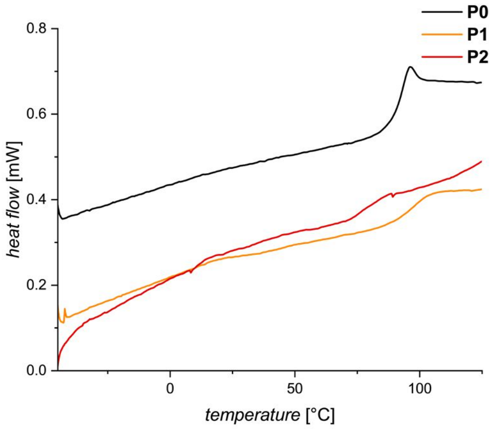

Figure S1. Differential scanning calorimetry (DSC) thermograms of **P0**, **P1**, and **P2.**

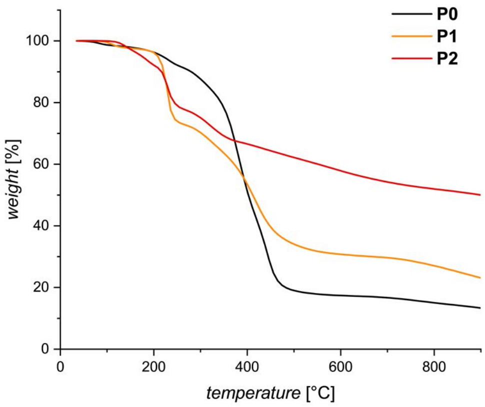

Figure S2. Thermogravimetric analysis curves of **P0**, **P1**, and **P2**.

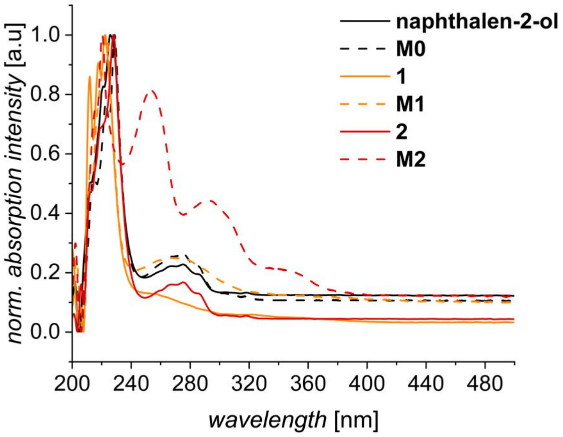

Figure S3. UV-Vis spectra of monomers (- - -) and their precursors (–).

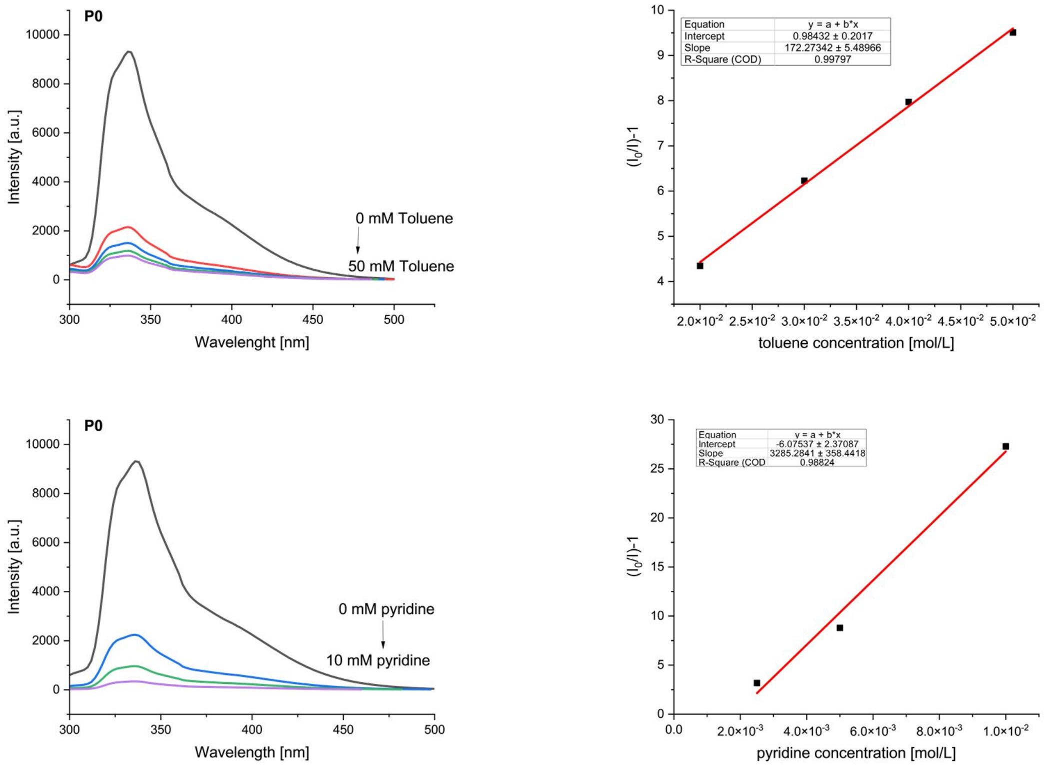

Figure S4. Fluorescence measurements for **P0** with increasing quencher concentrations and corresponding Stern-Volmer-plots with linear fit.

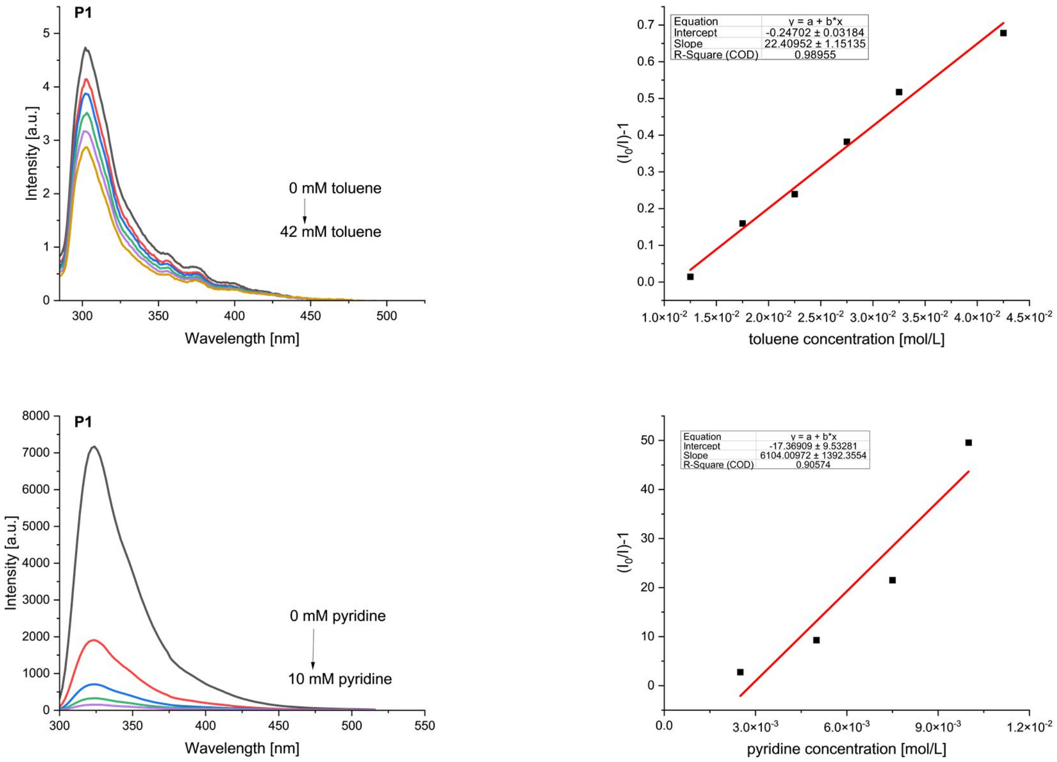

Figure S5. Fluorescence measurements for **P1** with increasing quencher concentrations and corresponding Stern-Volmer-plots with linear fit.

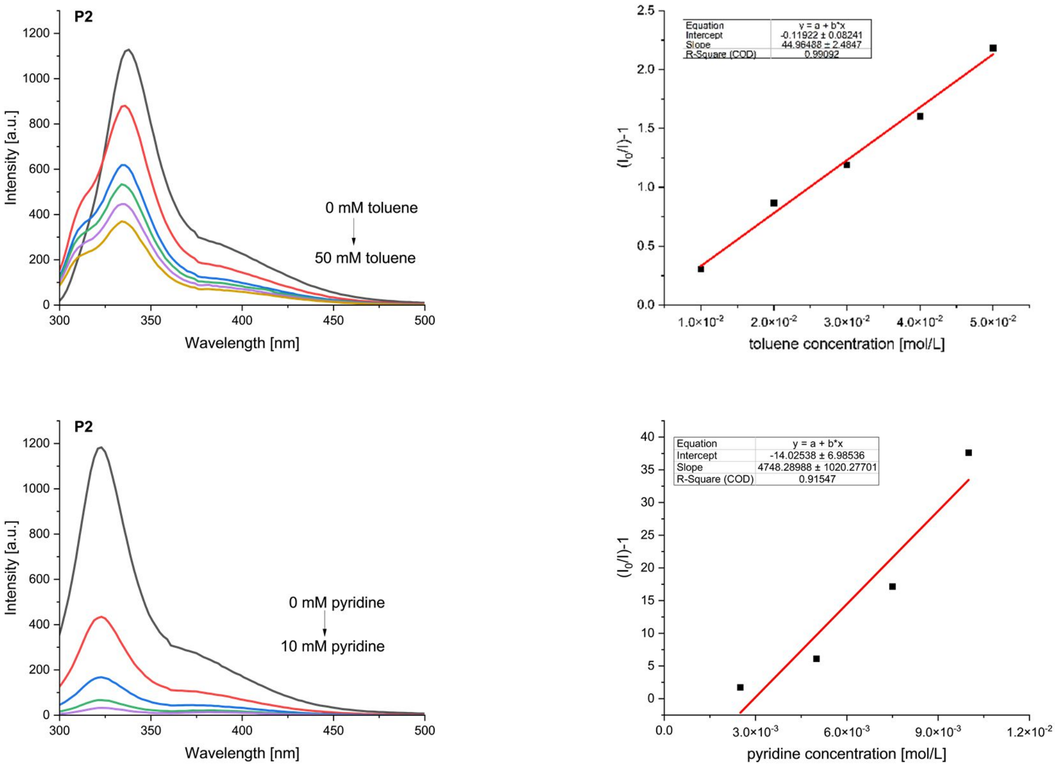

Figure S6. Fluorescence measurements for **P2** with increasing quencher concentrations and corresponding Stern-Volmer-plots with linear fit.

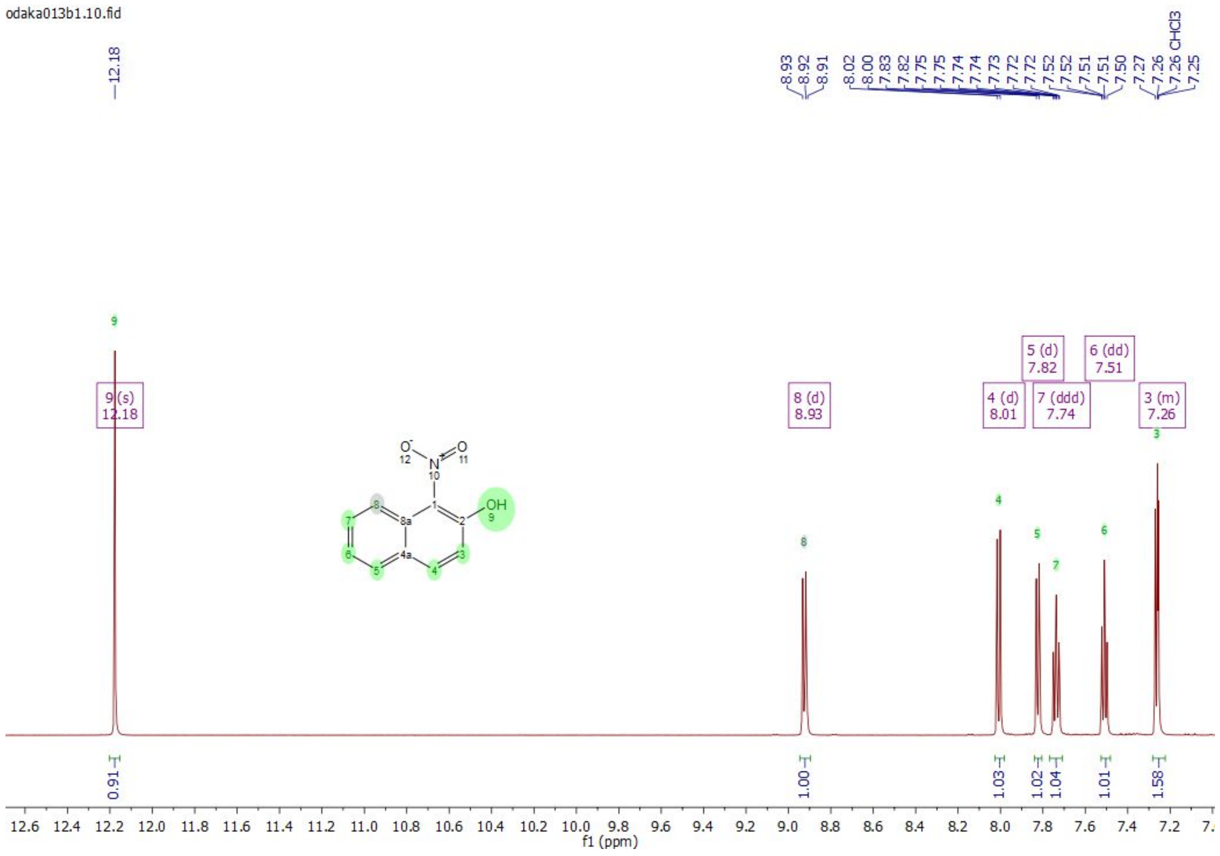

Figure S7. ^{1}H NMR spectrum of 1-nitronaphthalen-2-ol (**1**).

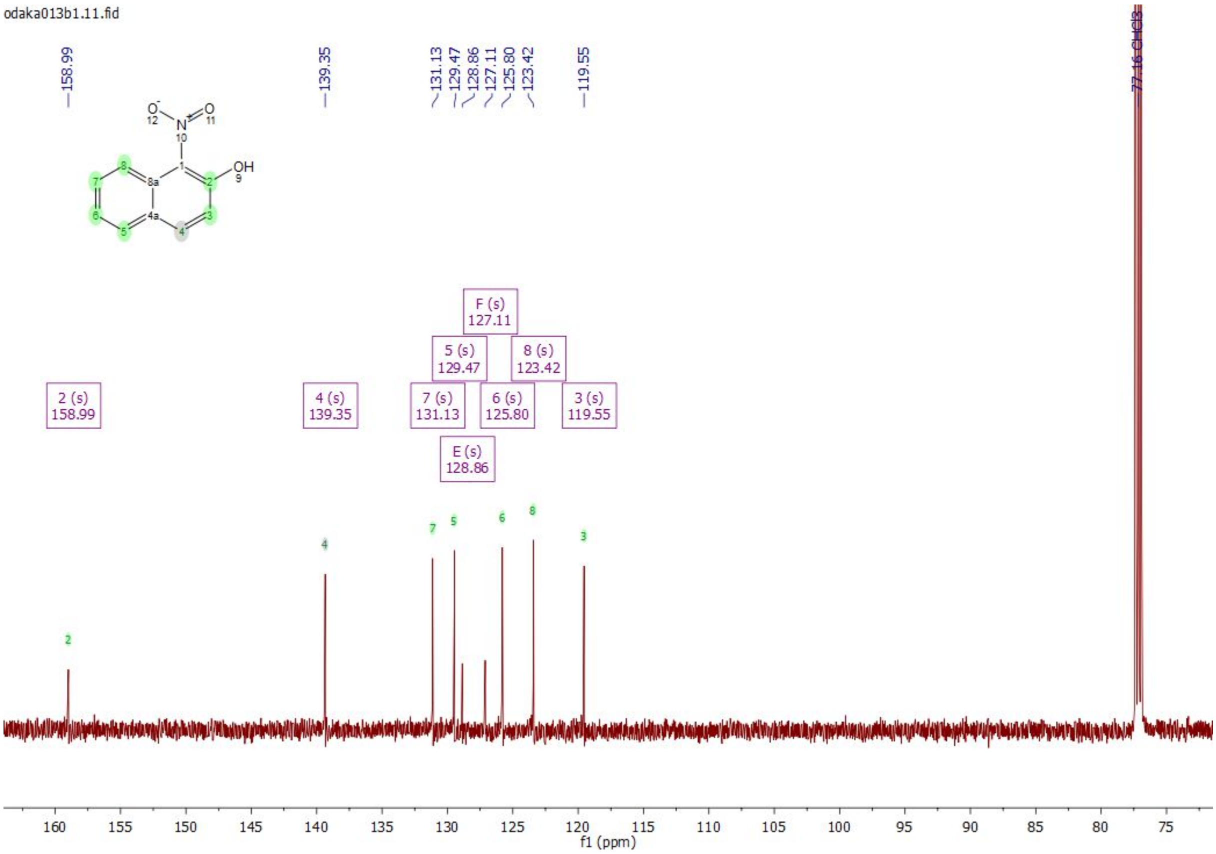

Figure S8. ^{13}C NMR spectrum of 1-nitronaphthalen-2-ol (**1**).

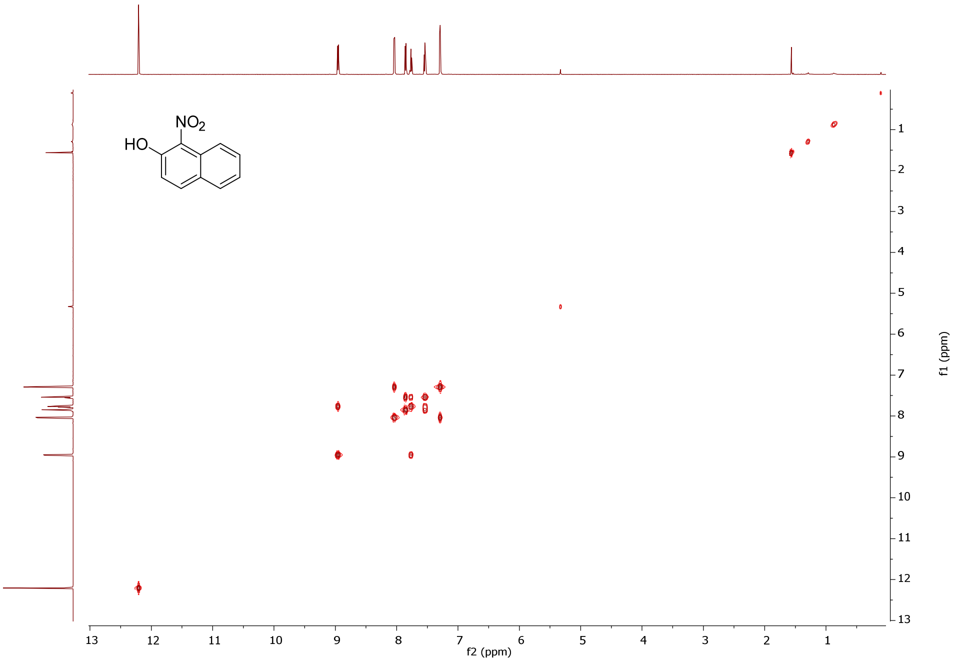

Figure S9. ^{1}H-^{1}H-Cosy NMR spectrum of 1-nitronaphthalen-2-ol (**1**).

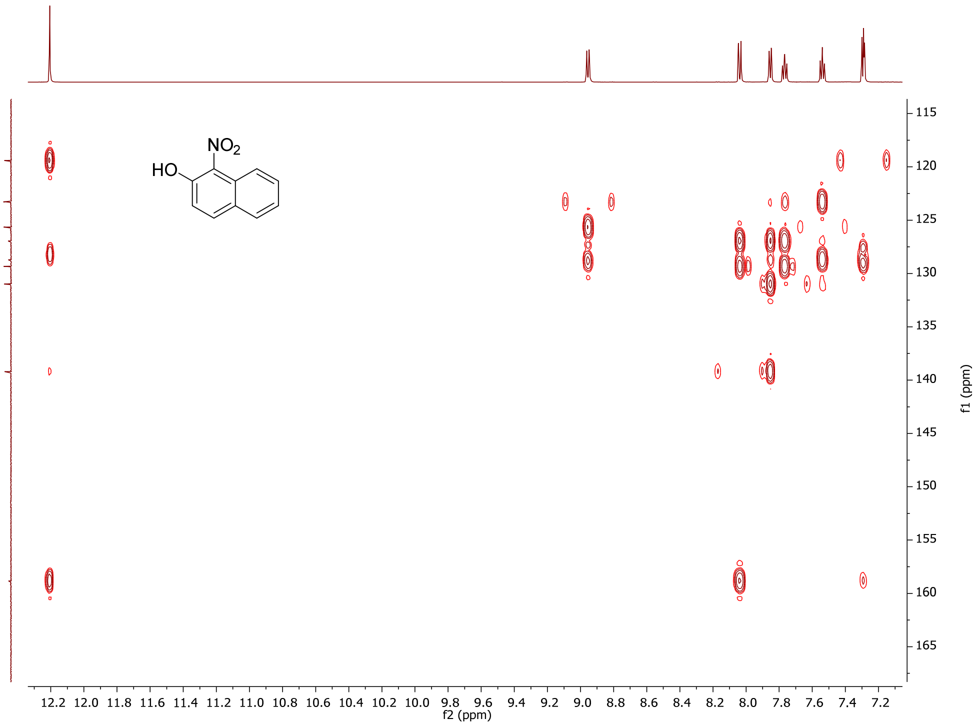

Figure S10. ^{13}C-^{1}H HMBC NMR spectrum of 1-nitronaphthalen-2-ol (**1**).

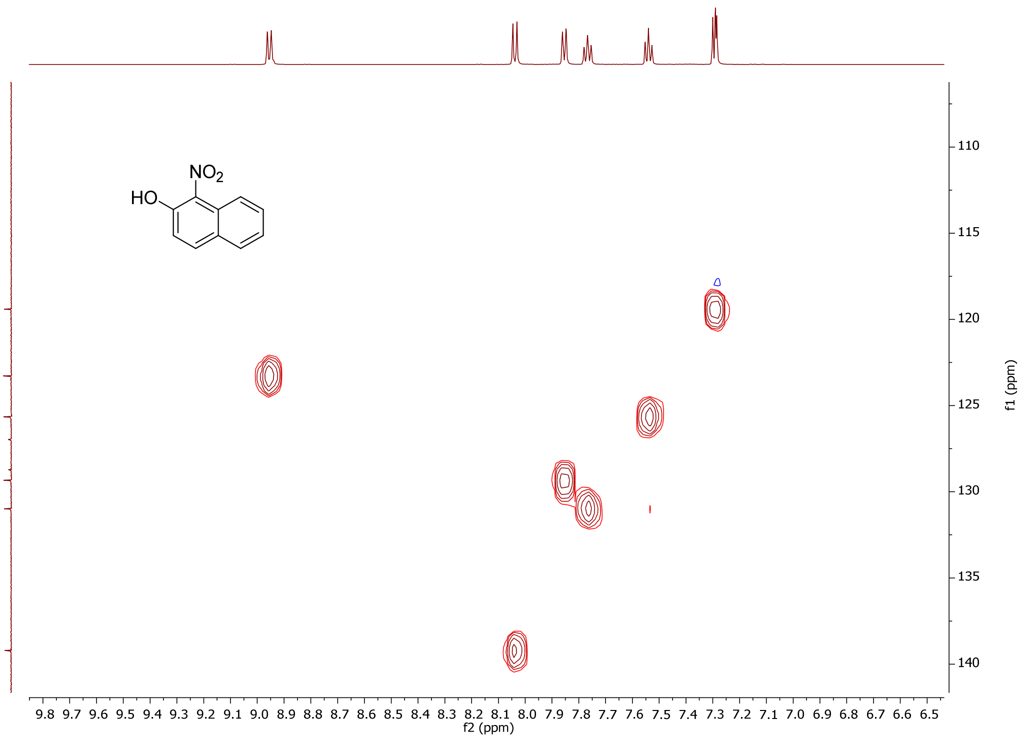

Figure S11. ^{13}C-^{1}H HSQC NMR spectrum of 1-nitronaphthalen-2-ol (**1**).

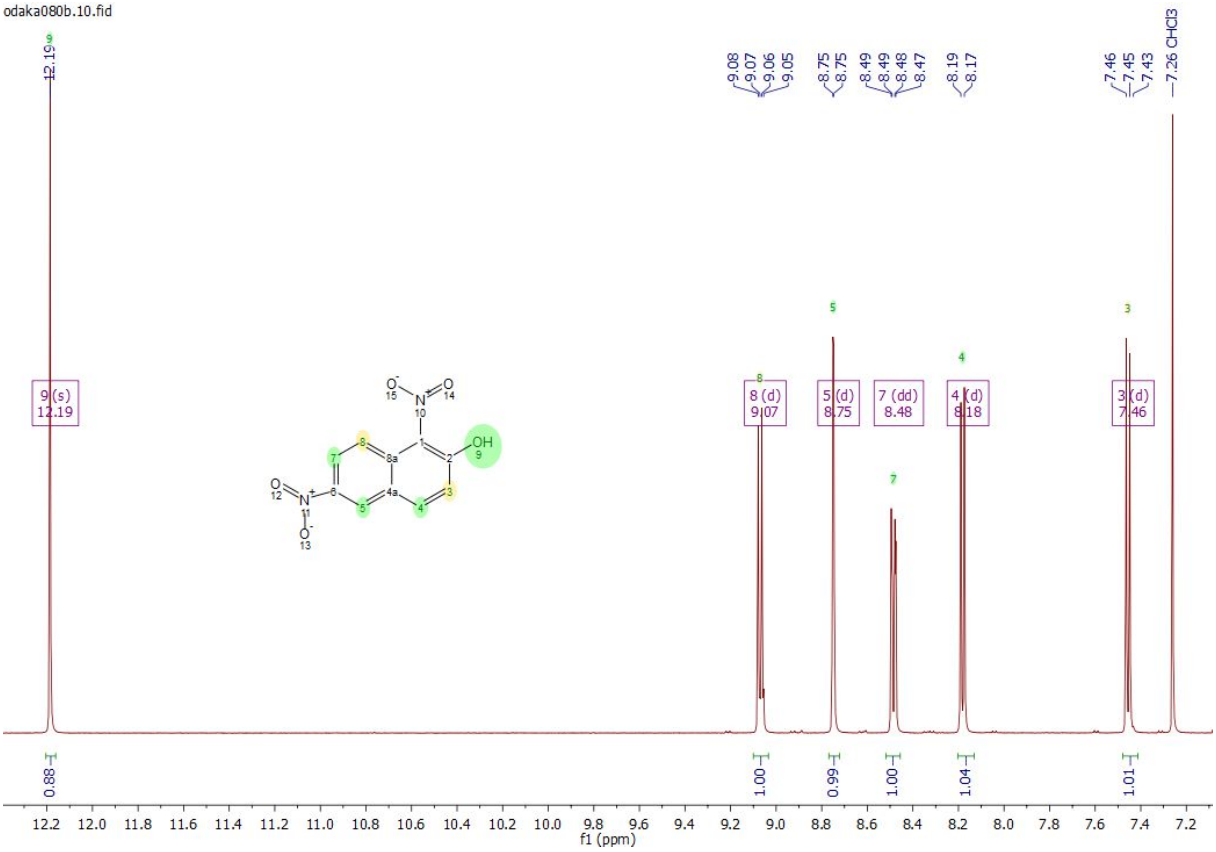

Figure S12. ^{1}H NMR spectrum of 1,6-dinitronaphthalen-2-ol (**2**).

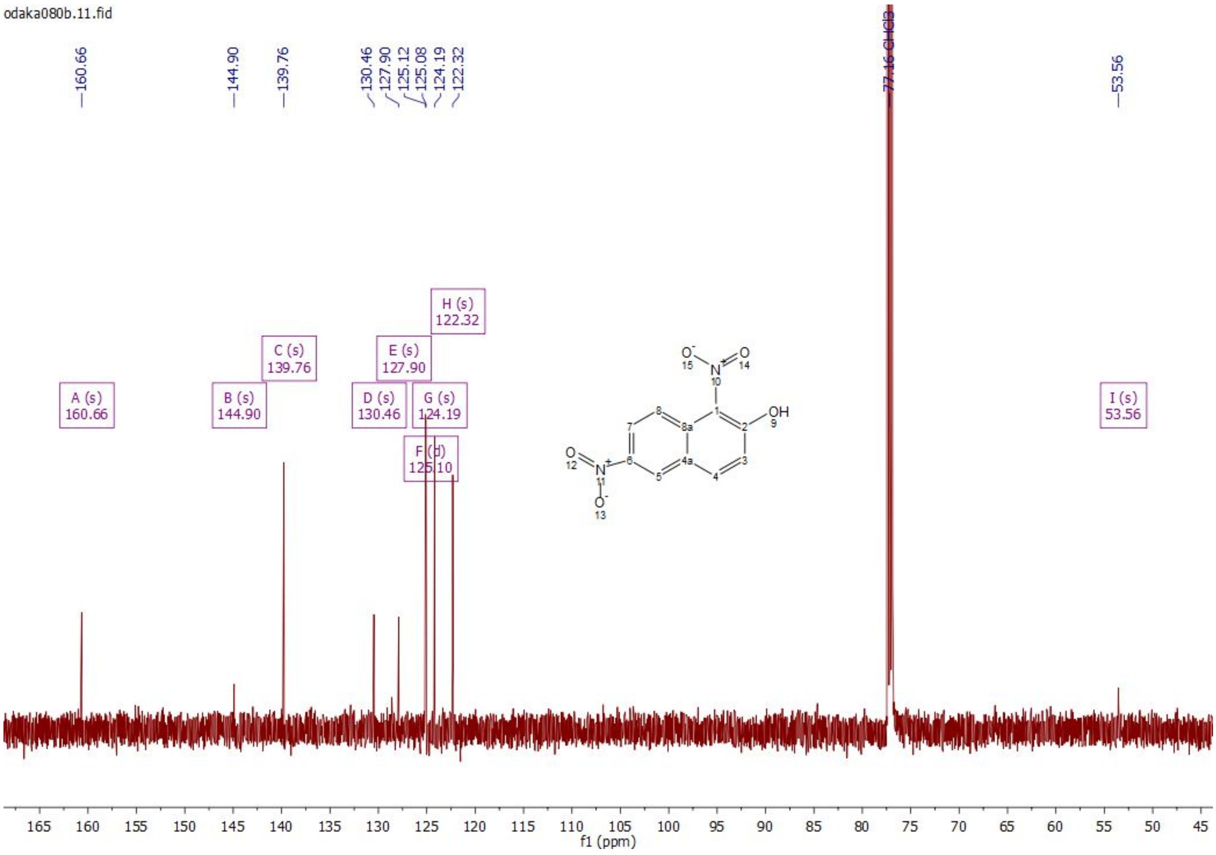

Figure S13. ^{13}C NMR spectrum of 1,6-dinitronaphthalen-2-ol (**2**).

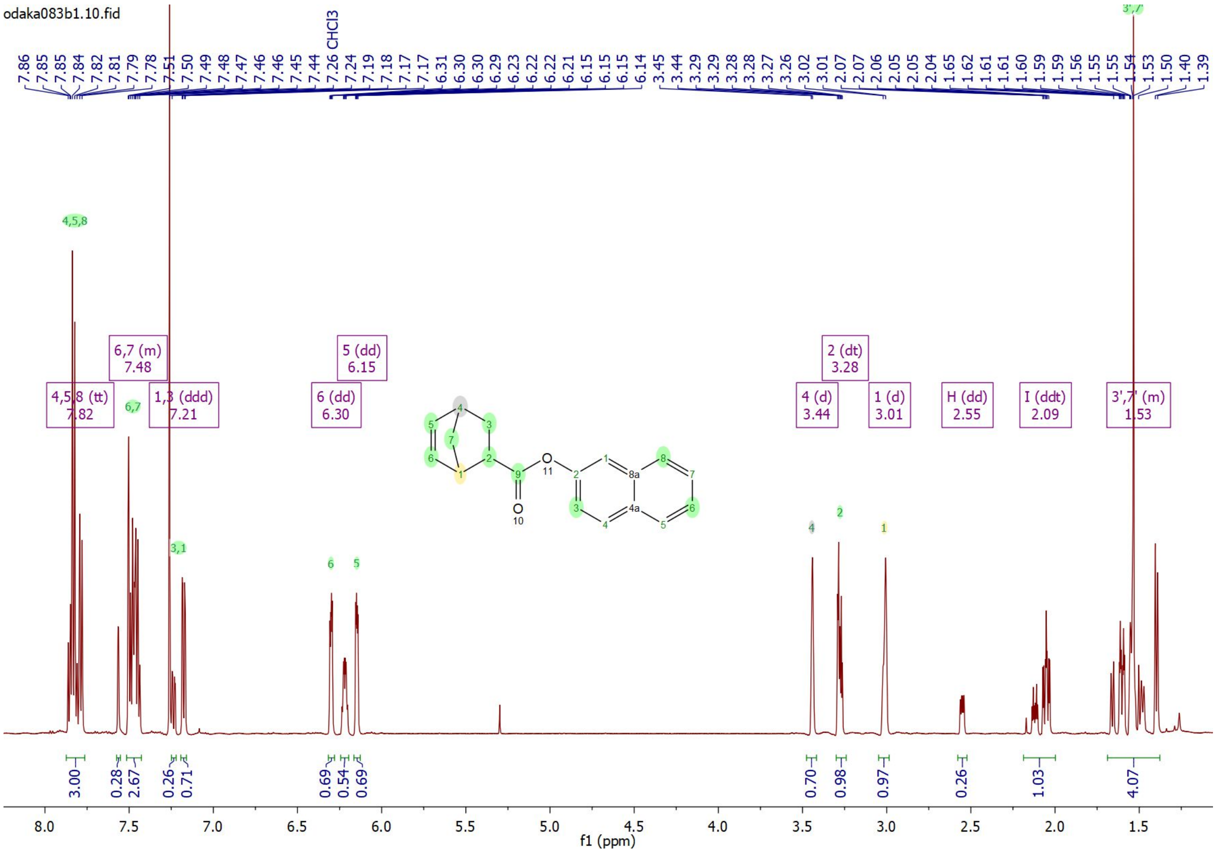

Figure S14. ^{1}H NMR spectrum of naphthalen-2-yl bicyclo[2.2.1]heptane-2-carboxylate (**M0**).

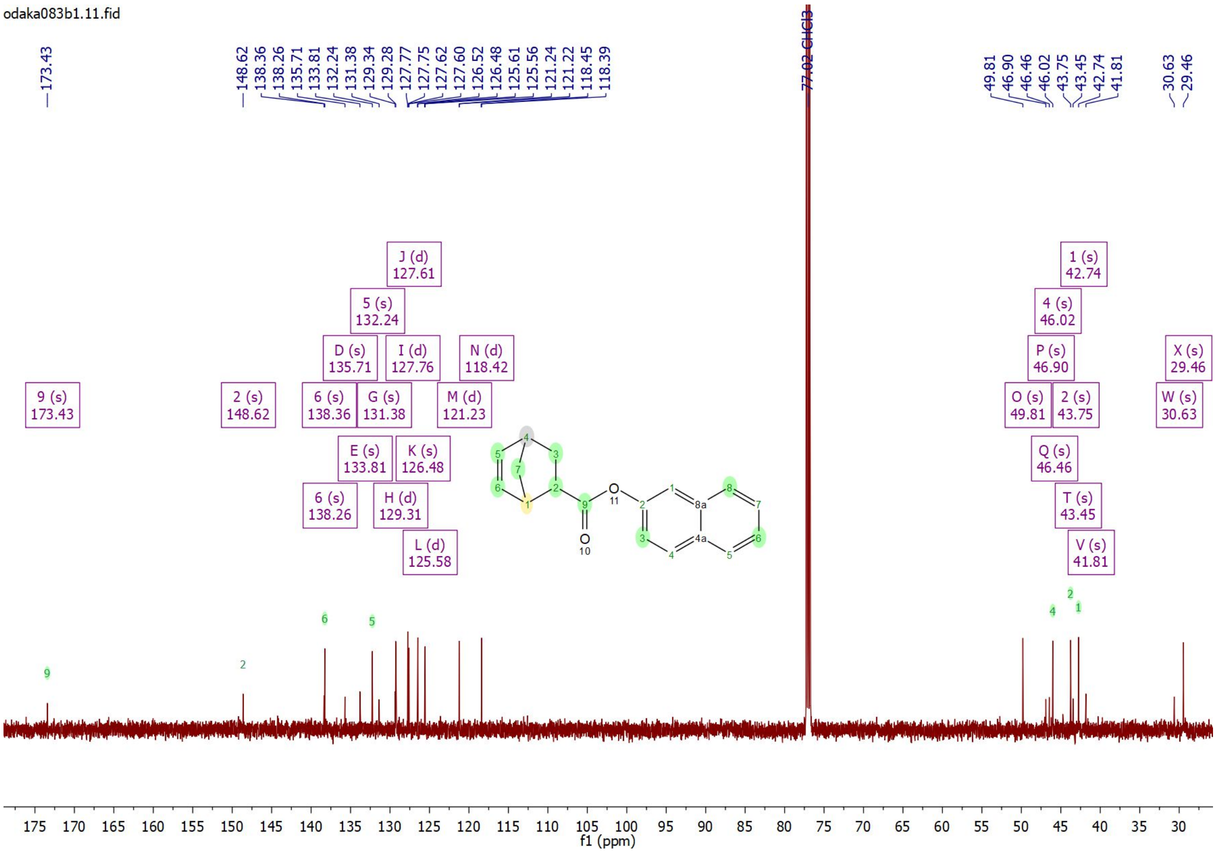

Figure S15. ^{13}C NMR spectrum of naphthalen-2-yl bicyclo[2.2.1]heptane-2-carboxylate (**M0**).

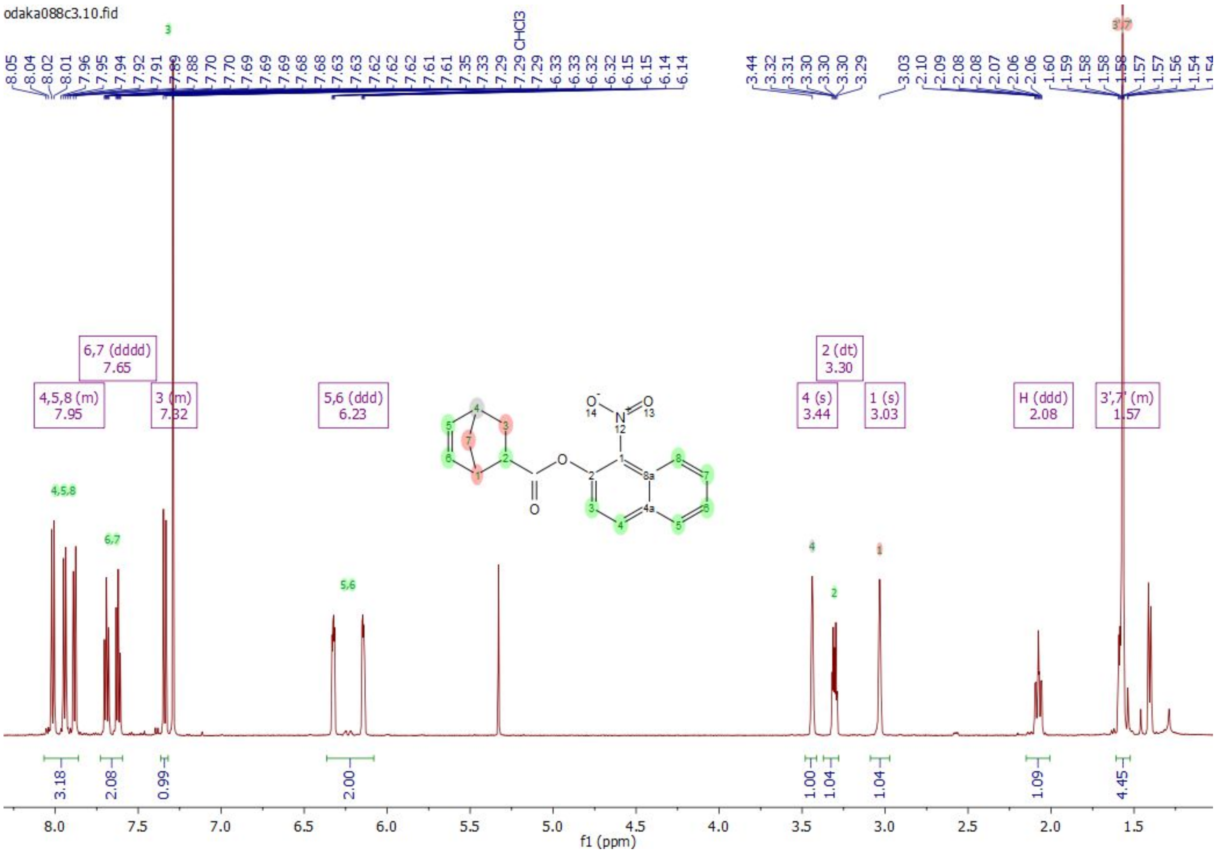

Figure S16. ^{1}H NMR spectrum of 1-nitronaphthalen-2-yl (1S)-bicyclo[2.2.1]hept-5-ene-2-carboxylate (**M1**).

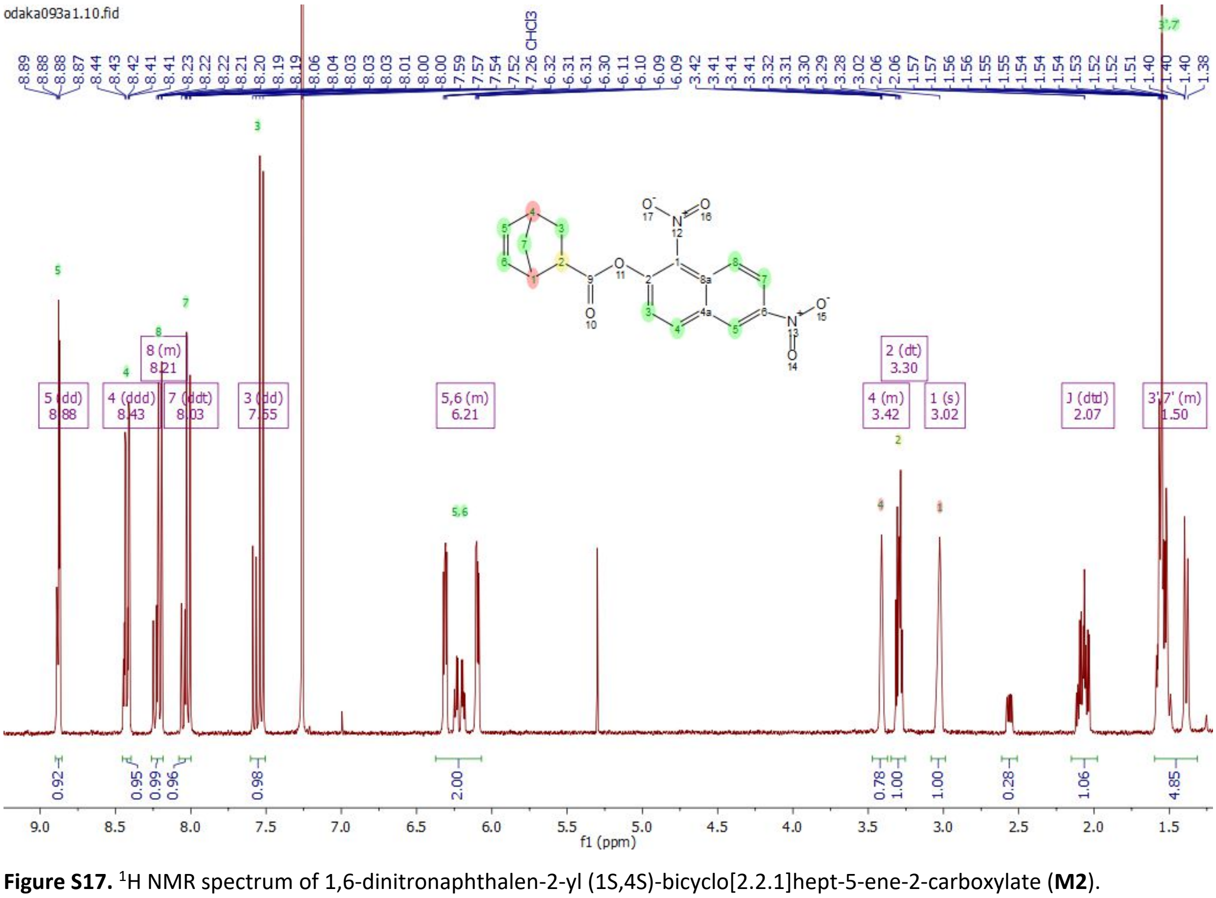

Figure S17. ^{1}H NMR spectrum of 1,6-dinitronaphthalen-2-yl (1S,4S)-bicyclo[2.2.1]hept-5-ene-2-carboxylate (**M2**).

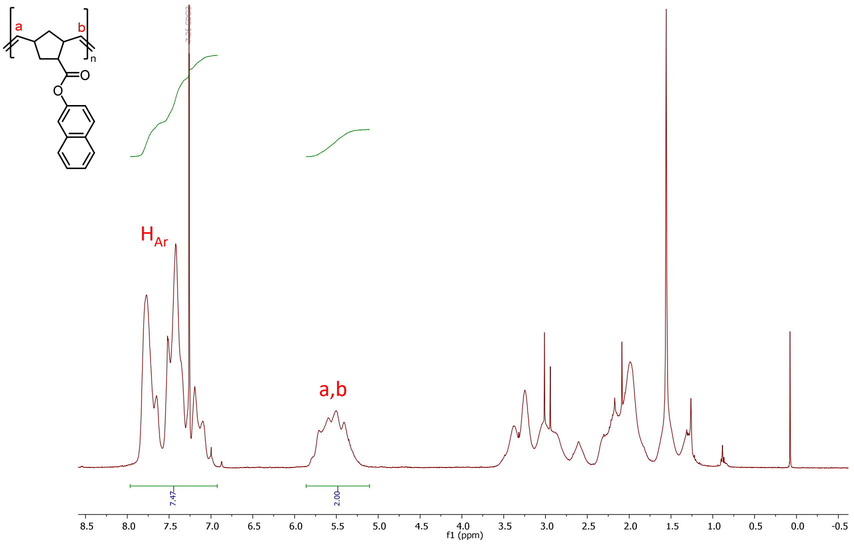

Figure S18. ^{1}H NMR spectrum of **P0**.

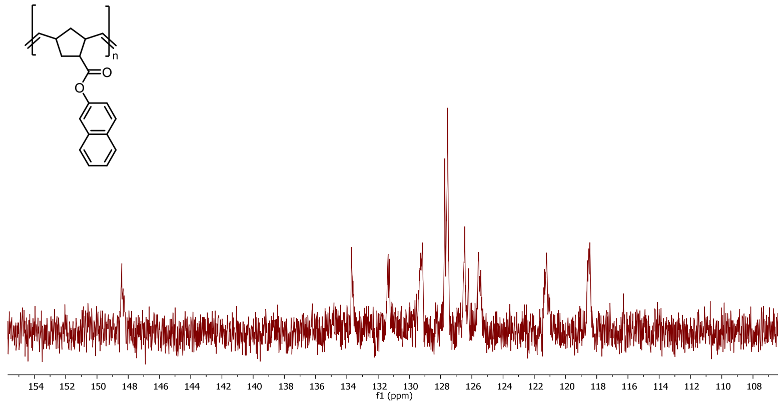

Figure S19. ^{13}C NMR spectrum of **P0**.

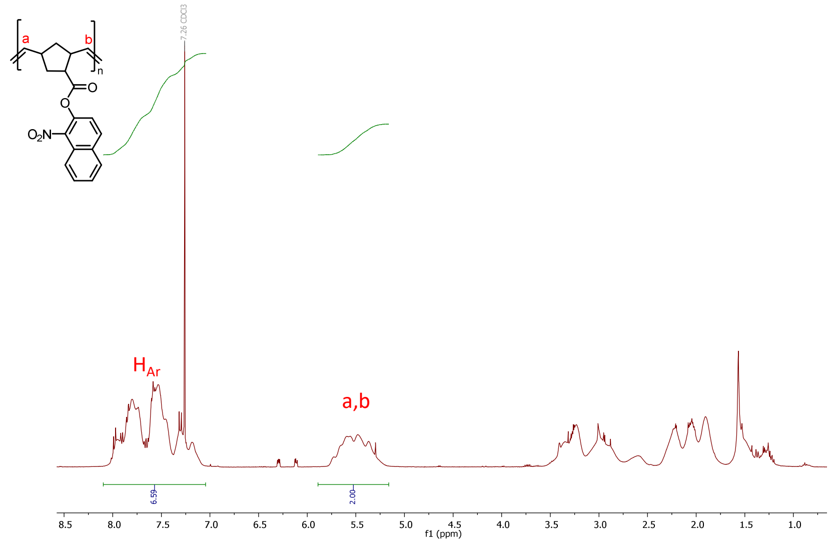

Figure S20. ^{1}H NMR spectrum of **P1**.

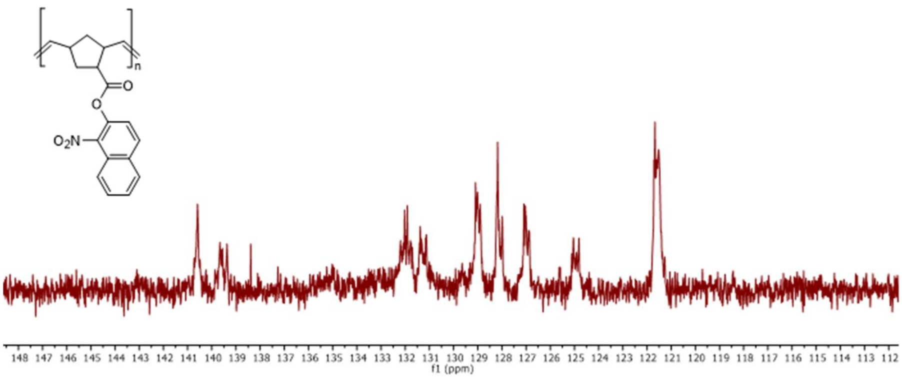

Figure S21.^{13}C NMR spectrum of **P1**.

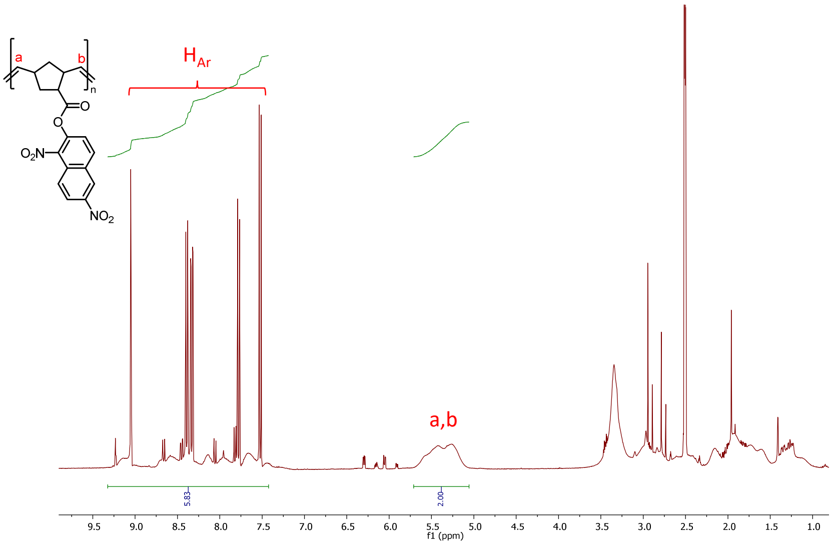

Figure S22. ^{1}H NMR spectrum of **P2**.

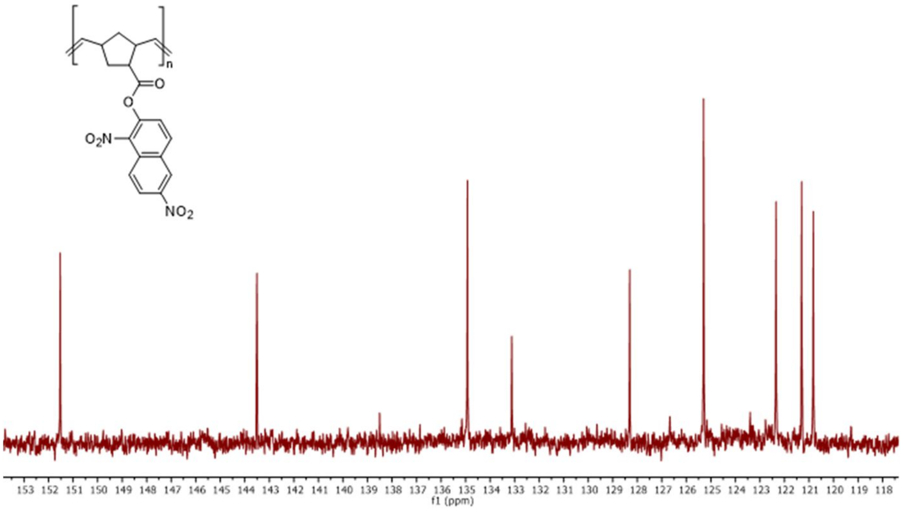

Figure S23. ^{13}C NMR spectrum of **P2**.
